# Laminin matrix regulates beta-cell FGFR5 expression to enhance glucose-stimulated metabolism

**DOI:** 10.1038/s41598-022-09804-7

**Published:** 2022-04-12

**Authors:** Vidhant Pal, Yufeng Wang, Romario Regeenes, Dawn M. Kilkenny, Jonathan V. Rocheleau

**Affiliations:** 1 Institute of Biomedical Engineering, University of Toronto, Toronto, Canada; 2 Toronto General Hospital Research Institute, University Health Network, Toronto, Canada; 3 ISTEP, University of Toronto, Toronto, Canada; 4 Department of Physiology, University of Toronto, Toronto, Canada; 5 Department of Medicine, University of Toronto, Toronto, Canada

**Keywords:** Fluorescent proteins, Type 2 diabetes, Endocrine system and metabolic diseases

## Abstract

We previously showed that pancreatic beta-cells plated on laminin matrix express reduced levels of FGFR1, a receptor linked to beta-cell metabolism and differentiation. Due to recent evidence that adult beta-cells also express FGFR5, a co-receptor for FGFR1, we now aim to determine the effect of laminin on FGFR5 expression and consequent effects on beta-cell metabolism. Using a genetically encoded sensor for NADPH/NADP^+^ redox state (Apollo-NADP^+^), we show overexpression of FGFR5 enhances glucose-stimulated NADPH metabolism in beta-cell lines as well as mouse and human beta-cells. This enhanced response was accompanied by increased insulin secretion as well as increased expression of transcripts for glycolytic enzymes (Glucokinase/GCK, PKM2) and the functional maturity marker Urocortin 3 (UCN3). Culturing beta-cells on laminin matrix also stimulated upregulation of endogenous FGFR5 expression, and similarly enhanced beta-cell glucose-stimulated NADPH-metabolism as well as GCK and PKM2 transcript expression. The metabolism and transcript responses triggered by laminin were disrupted by R5ΔC, a truncated receptor isoform that inhibits the FGFR5/FGFR1 signaling complex. Collectively these data reveal that beta-cells respond to laminin by increasing FGFR5 expression to enhance beta-cell glucose metabolism.

## Introduction

The biological markers that define beta-cell heterogeneity are still debated^1–3^ however it is recognized that the varied beta-cell microenvironment, specifically the deposition of ECM proteins within islets, could have a profound impact on beta-cell morphology, function, and genetic signature^4,5^. Previous work has shown that heterogeneous deposition of ECM by endothelial cells (ECs) and pericytes occurs throughout the 3D islet architecture^6,7^ and that dynamic remodeling of the microenvironment can alter and reorganize ECM composition to create a more suitable niche for islet cells^8,9^. With this in mind, matrix remodeling and ECM heterogeneity may play a key role in maintaining beta-cell plasticity that is critical during periods of proliferation, maturation, and stress^10^.

Expression of FGF ligands and FGFRs in embryonic pancreas^11^ and adult islets^12^ suggests regulation of FGF-mediated biological responses throughout the beta-cell life cycle. A role for FGFR1 activity in mature beta-cells was first demonstrated in a mouse model of dominant negative receptor expression driven by the PDX-1 promoter^13^. These mice developed a diabetic phenotype due to impaired Glut2 expression and increased pro-insulin content. We subsequently explored FGFR1 expression specifically in beta-cells and showed individual components of the beta-cell basement membrane (collagen type IV, laminin) differentially influence FGFR1 expression levels and consequent activation of relevant intracellular signaling pathways^14^. This study suggested the ECM microenvironment and in particular laminin regulates FGFR1 expression and activity in adult beta-cells. More recently we revealed beta-cell FGFR1 activity is also regulated by co-receptors such as Klothoβ (KLB) and FGFR5 (also identified as FGFRL1)^15,16^. Given that FGFR5 lacks the split intracellular tyrosine kinase domain exhibited by canonical FGF receptors, it has been identified as an inhibitor of cellular proliferation and a promoter of cellular adhesion and differentiation^17^. Our lab has further demonstrated FGFR5 expression to increase FGF2-induced ERK1/2 activation in beta-cells thus modulating, rather than blocking, downstream FGFR1 signaling^18^. Additionally, we have shown that FGFR5 expression is upregulated by cytokine induced stress and enhances beta-cell survival^16^. Given evidence that FGFR5 and FGFR1 form a co-receptor heterocomplex, our goal in this study was to determine the effect of FGFR5 expression on beta-cell metabolism and function. Previous work has highlighted the role of laminins in directing beta-cell maturation^19^. Given our prior work showing laminin matrix affects FGFR1 expression, we additionally aimed to determine whether laminin matrix modulates expression of FGFR5. Specifically, we hypothesized that laminin regulates FGFR5 expression and activity, thereby identifying differential laminin deposition in islets as a potential driver of heterogeneity in beta-cell responsiveness.

To better understand the effects of laminin on beta-cell FGFR5 expression and metabolism, we first imaged FGFR5-induced changes in metabolism using the genetically encoded Apollo-NADP^+^ sensor^20^. This strategy allowed us to uniquely measure changes in glucose-stimulated NADPH production in living beta-cells from cell lines as well as primary mouse and human islets. We also examined FGFR5-induced changes at the transcript level for genes associated with beta-cell metabolism and functional maturity. To explore the influence of the laminin matrix, we examined changes in endogenous FGFR5 expression and metabolic activity of beta-cells cultured on laminin compared to control cells cultured on Poly-D-Lysine (PDL). We further explored disrupting the FGFR5/FGFR1 signaling complex by introducing a truncated FGFR5 receptor construct (R5ΔC), as well as the effects of complex disruption on FGFR5- and laminin-induced metabolism. Finally, we discuss our data in the context of varied laminin deposition and ECM remodeling, connecting our work to the broader discussion surrounding the ability of the islet microenvironment to regulate beta-cell heterogeneity.

## Results

### FGFR5 expression in beta-cell lines enhances glucose-stimulated metabolic responses

**Figure 1:**
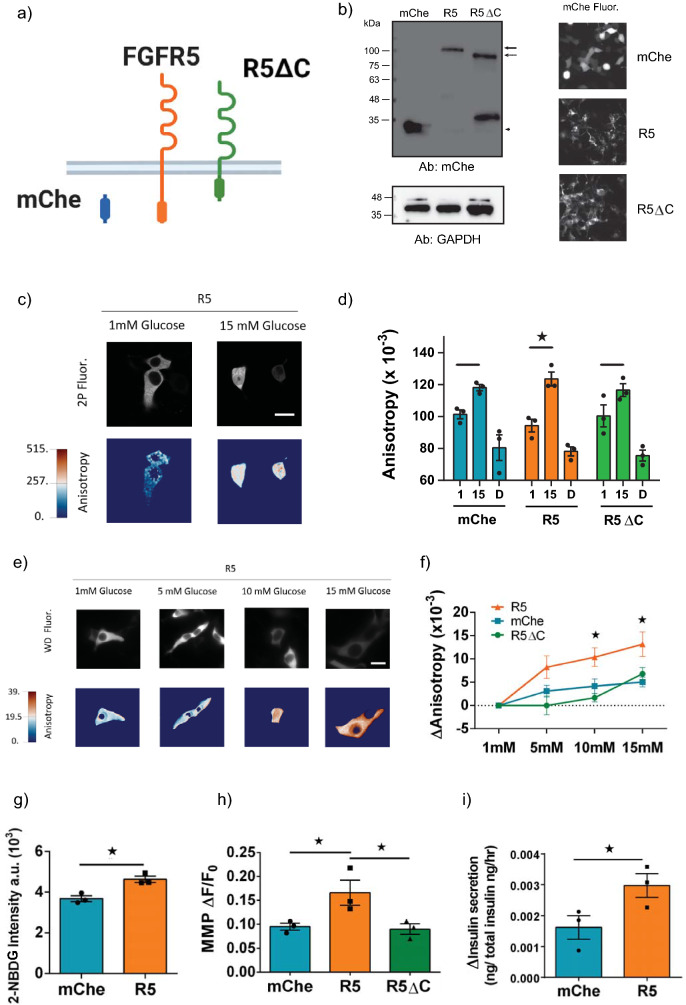
R5 overexpression induces a more robust glucose-stimulated metabolic response in beta-cell lines. To explore the effect of FGFR5 on beta-cell metabolism, we overexpressed mCherry (mChe), mCherry-tagged FGFR5 (R5), or mCherry-tagged C-terminal truncated FGFR5 (R5ΔC) in beta-cell lines and tested a variety of metabolic parameters. (**a**) Cartoon comparing the constructs relevant to these studies. (**b**) The full-length R5 construct was identified as a protein band with molecular mass of ~ 105–110 kDa (solid arrow). The truncated R5ΔC construct was identified as a protein band with molecular mass of ~ 85–90 kDa (dashed arrow) while the mChe was identified as a protein band with molecular mass ~ 28 kDa (arrowhead). Construct expression within beta-cell cultures was confirmed by mChe immunofluorescence (representative images shown at right). (**c**) Representative 2-photon fluorescence (2P Fluor) and anisotropy images of the genetically encoded Apollo NADP^+^ sensor expressed in INS1E cells and in response to 1 mM and 15 mM glucose (scale bar = 5 μm; anisotropy lookup table for 2P =  × 10^–3^). (**d**) INS1E cells expressing the Apollo-NADP^+^ sensor and either mChe, R5, or R5ΔC were exposed to 1 mM and 15 mM glucose, followed by 2 mM diamide to deplete NADPH (sensor anisotropy reduced to baseline). (**e**) Representative widefield fluorescence (WD Fluor) and anisotropy images of the genetically encoded Apollo NADP^+^ sensor expressed in βTC3 cells at multiple glucose concentrations, as indicated (scale bar = 5 μm; anisotropy lookup table for widefield anisotropy =  × 10^–2^). (**f**) Anisotropy response of βTC3 cells overexpressing R5, mChe, and R5ΔC in response to sequential glucose concentrations, as indicated. (**g**) βTC3 cells overexpressing mChe or R5 were co-incubated with 15 mM glucose and 2 µM 2-NBDG for 20 min at 37 °C prior to fluorescence imaging. (**h**) βTC3 cells expressing mChe, R5, or R5ΔC were labelled in 1 mM glucose supplemented with 5 µM Rh123 for 7 min to achieve dye self-quenching. Data represents the relative change in fluorescence intensity (ΔF/F_0_) in response to a bolus of 15 mM glucose. (**i**) βTC3 cells overexpressing mChe or R5 were incubated at 1 mM and 15 mM glucose to test glucose stimulated insulin secretion response. Data represent the difference in fractional insulin secretion in response to 1- and 15-mM glucose. Bars and line graphs show the average ± SEM of n = 3 independent experiments. *, *p* < 0.05 using ANOVA followed by one-tailed t-test.

Glucose triggers insulin secretion through generation of NAD(P)H resulting in sequential production of ATP, closure of K_ATP_ channels, and Ca^2+^ influx^21^. To measure the effect of FGFR5 on beta-cell metabolism, we overexpressed fluorescently tagged FGFR5 constructs in beta-cell lines and measured changes in glucose-stimulated NADPH metabolism, glucose-transport, mitochondrial membrane potential, and insulin secretion (Fig. 1). The FGFR5 constructs included a full-length FGFR5-mCherrry (R5), C-terminally truncated FGFR5 receptor (R5ΔC), and mCherry control (mChe) (Fig. 1a). Construct expression was confirmed by mCherry Western immunoblotting and fluorescence imaging (Fig. 1b, S1). A band of ~ 105–110 kDa was detected exclusively in the R5 sample, consistent with the molecular mass anticipated for FGFR5 protein with an mCherry tag and considering glycosylation effects. A smaller band of ~ 85–90 kDa was observed in the R5ΔC sample, as expected in the absence of the C-terminal tail. The mChe sample exhibited a unique band at the expected molecular mass of ~ 28 kDa. Comparison of band intensities to the GAPDH housekeeping protein suggests similar construct expression levels (Fig. 1b, left panels) that are reflected by live cell imaging of the relevant cell cultures (Fig. 1b, right panels). We first measured the glucose-stimulated NADPH response in INS1E cells using the anisotropy-based Apollo-NADP^+^ sensor co-transfected with R5 (Fig. 1c). Glucose-stimulated NADPH responses in the cytoplasm occur primarily due to a metabolic route involving pyruvate entry into the tricarboxylic acid (TCA) cycle via anaplerosis^22^. These data show R5 expression enhanced glucose-stimulated NADPH response (i.e., greatest range in anisotropy measurements between 1 and 15 mM treatments) compared to cells expressing C-terminally truncated receptor (R5ΔC) or the mCherry vector control, which exhibited similar responses (Fig. 1d, S2). To confirm this R5-mediated effect in a different beta-cell line, we subsequently measured the glucose-stimulated NADPH response in βTC3 cells (Fig. 1e–f). These data show a more robust glucose-stimulated NADPH response (i.e., larger ΔAnisotropy) across the range of glucose treatments. To further explore the impact of R5 on βTC3 cell metabolism and function, we examined glucose-stimulated glycolytic flux using 2-NBDG, mitochondrial membrane potential using Rh123, and fractional insulin secretion by ELISA (Fig. 1g–i, S3). These data show R5 overexpression enhances each of these responses consistent with increasing glucose-stimulated metabolism and function in beta-cell lines. Notably, expressing a truncated FGFR5 construct deficient for the C-terminus (R5ΔC) did not result in significant responses compared to mChe, consistent with a dependence on the intracellular domain for proper receptor function.

### FGFR5 induces glucose-stimulated metabolism in primary mouse beta-cells

**Figure 2:**
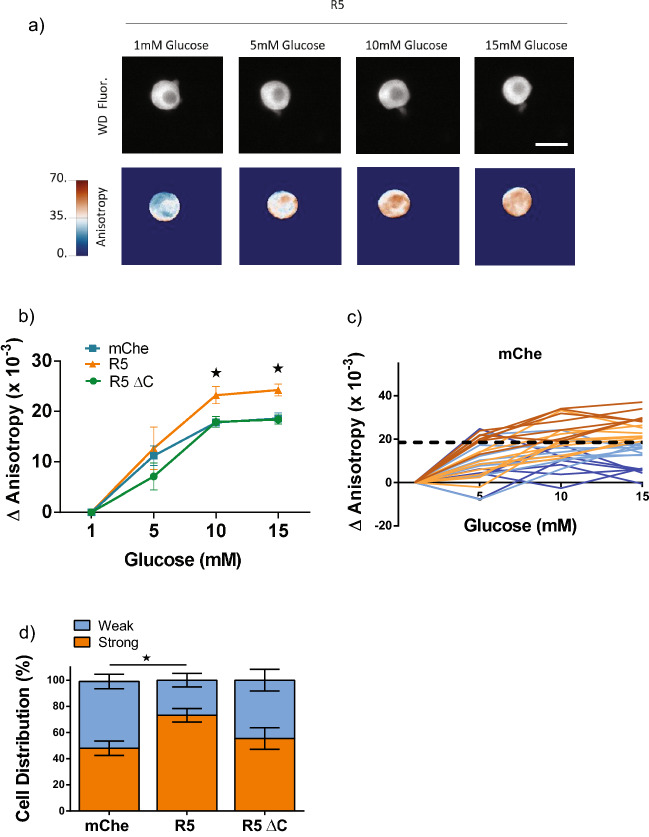
FGFR5 overexpression in primary mouse beta-cells induces a more robust glucose stimulated NADPH response​. To confirm the FGFR5-induced metabolic effects in primary tissue, R5 was overexpressed in murine beta-cells and the metabolic response observed by live cell imaging. Mouse islets were dispersed to single cells onto glass bottom dishes and co-transduced with the Apollo-NADP^+^ sensor using a CMV promoter and one of the mCherry constructs (R5, R5ΔC, or mChe; as indicated) using a rat insulin promoter. (**a**) Widefield fluorescence (WD Fluor) and anisotropy images from the same representative beta-cell exposed to multiple glucose concentrations, as indicated. (Scale bar = 5 μm; anisotropy lookup table for widefield anisotropy =  × 10^–2^). (**b**) Apollo-NADP^+^ response **(**ΔAnisotropy) of mCherry positive beta-cell cells overexpressing R5, mChe, and R5ΔC in response to sequential glucose concentrations, as indicated. (**c**) Traces of individual Apollo-NADP^+^ responses collected across three separate days are represented for mChe-expressing beta-cells. The lines are color-coded based on being above (orange-brown) or below (light blue-dark blue) the average 15 mM glucose response (ΔAnisotropy represented by the hatched black line = 19 × 10^–3^). Different shades were used to indicate when the magnitude of response was above (orange) and below (dark blue) the mean response. (**d**) The Apollo-NADP^+^ responses of R5 and R5ΔC expressing beta-cells (traces shown in Fig. S3) were classified as either strong (orange) or weak (dark blue) glucose responders based on the mean value defined by the mChe cells. Bars indicate % cell distribution for each construct. Bars graph shows the average ± SEM of n = 3 independent experiments. *, *p* < 0.05 using ANOVA followed by one-tailed t-test.

To confirm the effect of FGFR5 in a more physiologically relevant context, we measured the metabolism of beta-cells from mouse islets (Fig. 2). Mouse islets were dispersed onto poly-D-lysine coated dishes and co-transduced with the Apollo-NADP^+^ sensor under the CMV promoter and one of the R5 constructs (R5, R5ΔC, or mChe; as indicated) under a rat-insulin promoter (RIP). The NADPH responses (anisotropy images) were collected on a single cell basis (Fig. 2a). These data show a significantly greater NADPH-response in R5-expressing cells at 10 and 15 mM glucose compared to mChe and R5ΔC-expressing cells (Fig. 2b). The average final dose response of the wild-type control beta-cells (dotted black line) was used to define two sub-populations identified as high (*orange*) and low (*blue*) responders (Fig. 2c; Fig. S4). The mChe control cells showed a wide range of glucose-stimulated responses consistent with significant beta-cell metabolic heterogeneity. We subsequently used this threshold ΔAnisotropy to classify the fraction of high and low responders of R5 and R5ΔC expressing beta-cells (Fig. 2d, Fig. S4). These data show an increase in the fraction of high responders in R5 expressing cells compared to both mChe and R5ΔC expressing cells. Additionally, the NADPH response was proportional to the R5-construct expression, as measured by mCherry fluorescence intensity, in contrast to R5ΔC and mChe control (Fig. S5). Collectively, these data confirm that expression of FGFR5 proportionally enhances murine beta-cell metabolic responses relative to the degree of receptor expression.

### FGFR5 enhances expression of genes associated with glycolytic flux and late-stage markers of functional maturity

**Figure 3:**
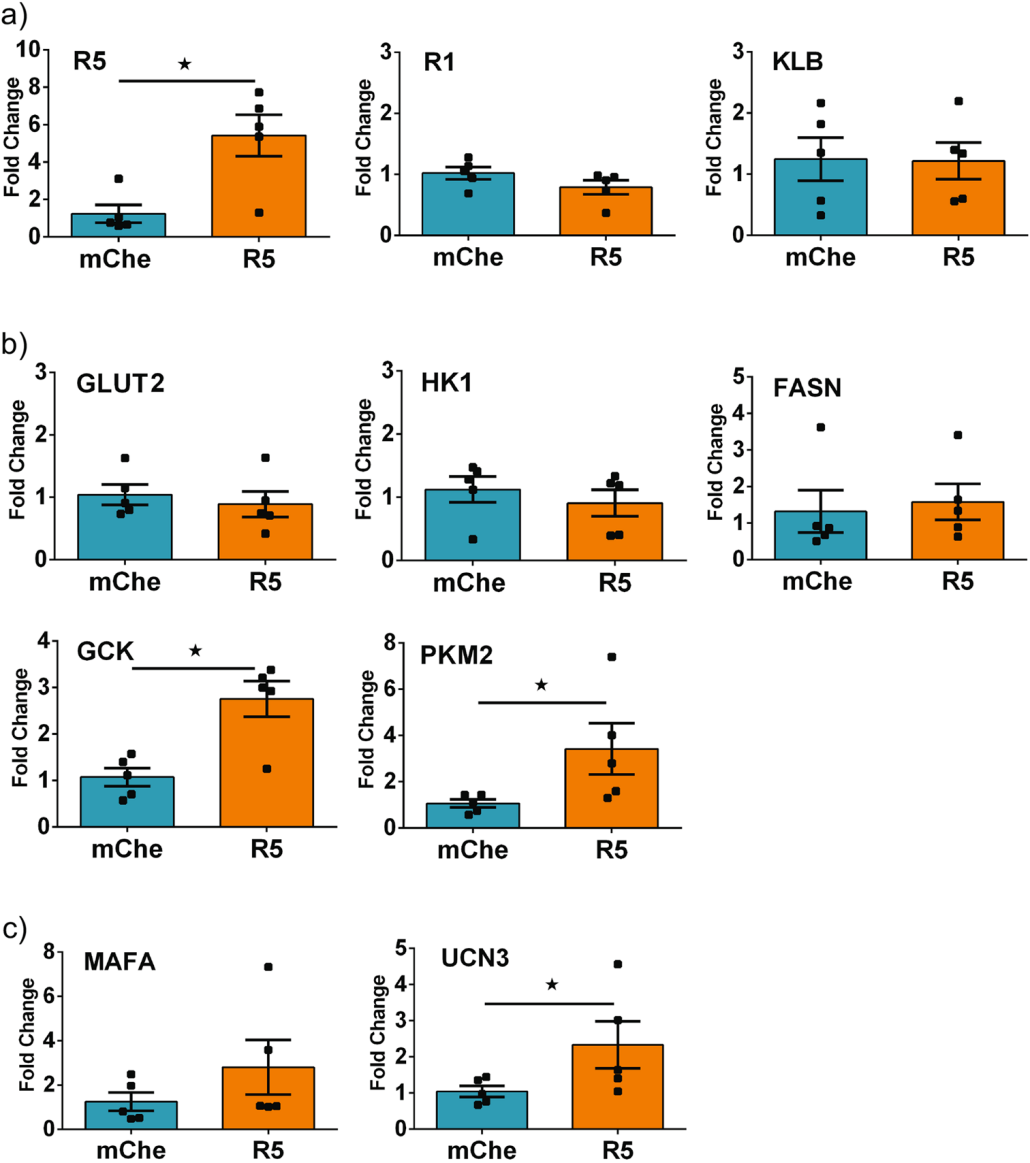
FGFR5 overexpression enhances levels of gene transcripts associated with glycolytic metabolism and maturity. To better understand the mechanism underlying FGFR5 induced metabolic remodeling, cell lines overexpressing R5 were examined for changes in transcript level of genes related to metabolism and maturity. βTC3 cells overexpressing mChe and R5 constructs were compared for transcript expression levels by qPCR. Transcript data is organized by: (**a**) FGF-associated receptors (FGFR5 (R5), FGFR1 (R1), KLB); (**b**) metabolic genes (GLUT2, HK1, FASN, GCK, PKM2); and (**c**) maturity markers (MAFA, UCN3). Bars indicate the average fold change in transcript level ± SEM; symbols represent the individual responses of n = 5 independent experiments. *, *p* < 0.05 using one-tailed t-test.

To better understand the mechanisms of FGFR5-induced metabolism, we measured changes in transcript expression in βTC3 cells overexpressing the R5 construct (Fig. 3). We first examined transcripts encoding relevant membrane receptors including FGFR5, FGFR1, and KLB (Fig. 3a). The observed fivefold increase in R5 transcript level was consistent with construct overexpression. In contrast, no measurable differences were observed in FGFR1 or KLB transcript levels. To further discern the effect of FGFR5 on metabolism, we measured changes in genes associated with glycolysis and fatty acid synthesis (Fig. 3b). No changes were observed in the level of transcripts encoding the glucose transporter (GLUT2), low-capacity hexokinase-1 (HK1), or fatty acid synthase (FASN) when R5 was overexpressed. In contrast, a significant increase in expression was observed for GCK and PKM2, suggesting R5 expression is associated with glucose sensing and pyruvate metabolism in mature beta-cells, *respectively*. To assess the role of FGFR5 on beta-cell maturation, we examined early (MAFA) and late-stage (UCN3) transcript markers^23,24^ (Fig. 3c). These data show a trend towards increased MAFA transcript and a statistically significant increase in UCN3 transcript level when R5 was overexpressed. Collectively, these data suggest that FGFR5 expression induces a more robust metabolic phenotype (GCK, PKM2) consistent with metabolic maturation (MAFA, UCN3) in beta-cell lines.

### Laminin matrix increases endogenous FGFR5 expression and glycolytic metabolism in beta-cell lines

**Figure 4:**
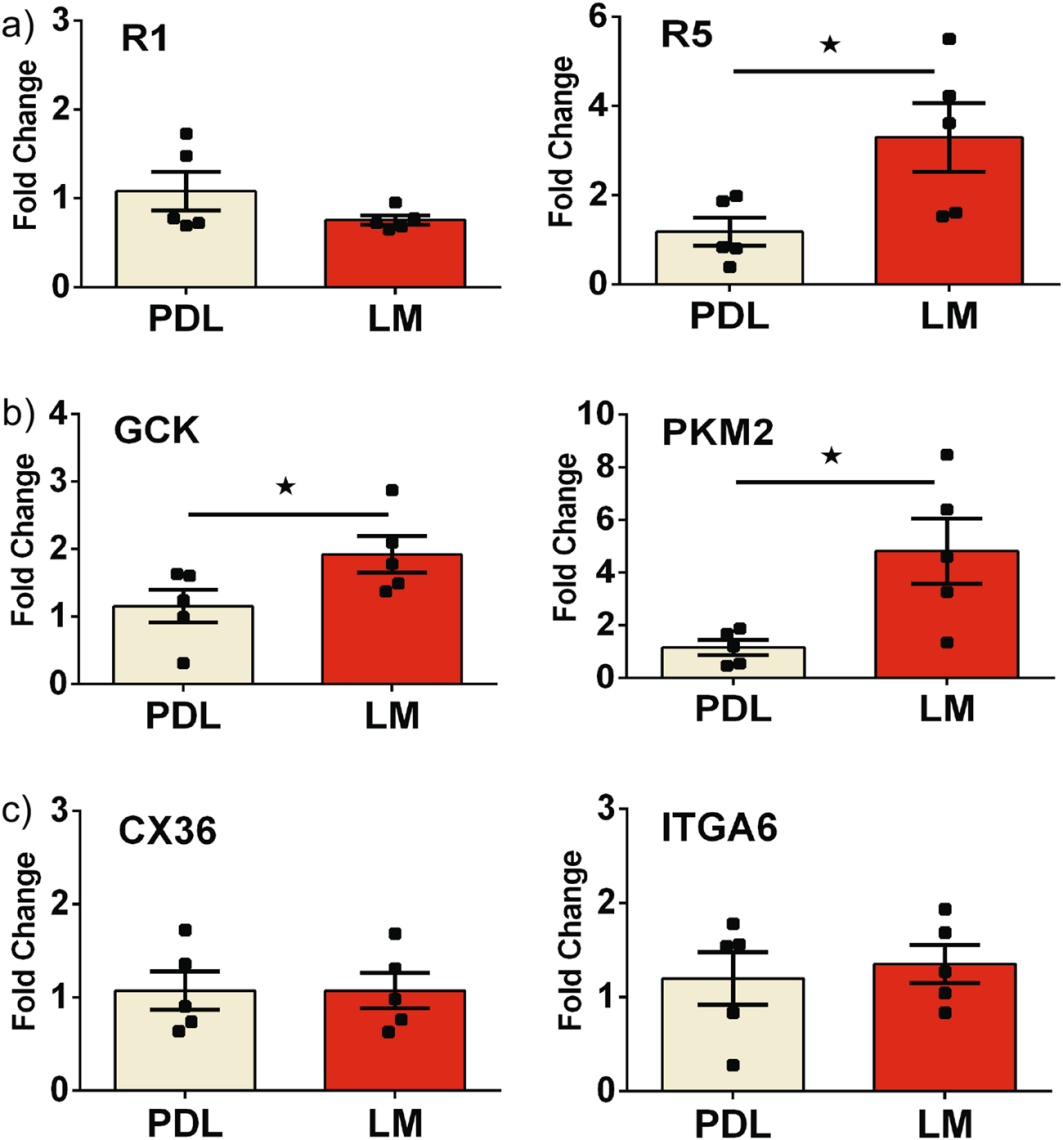
Culturing beta-cell lines on laminin matrix increases expression of FGFR5 transcript and glycolysis genes. Our previous work shows that the expression of FGFR5 coreceptor, FGFR1, is regulated by laminin matrix. To test whether laminin impacts expression of FGFR5 in beta-cells lines, βTC3 cells were plated on poly-D-lysine (PDL) or laminin matrix (LM) overnight for transcript expression analysis by qPCR. Data are organized by: (**a**) FGF-associated receptors (FGFR1 (R1), FGFR5 (R5)); (**b**) glycolytic metabolism (GCK, PKM2); and (**c**) cell adhesion/connectivity markers (CX36, ITGA6). Bars show the average fold-change in transcript level ± SEM; symbols represent the individual responses of n = 5 independent experiments. *, *p* < 0.05 using one-tailed t-test.

It has been established that beta-cells show increased functional maturity with adhesion to laminin, an ECM basement membrane component found in pancreatic islets primarily around mature blood vessels^25^. We previously showed that cell-surface FGFR1 expression levels are reduced when beta-cells are cultured on laminin matrix^14^ while FGFR5 overexpression increased beta-cell adhesion to the culture matrix^18^. To further elucidate the link between beta-cell ECM and FGFR5 activity, we used qPCR to examine endogenous transcript levels of FGF receptors, cell adhesion and connectivity proteins, and metabolic genes previously identified (Fig. 4). Compared to cultures on PDL, βTC3 cells plated on laminin matrix exhibited a trend towards decreased FGFR1 transcript expression and significantly increased FGFR5 transcript expression, suggesting a potential increase in the FGFR5:FGFR1 ratio (Fig. 4a). These data also showed a laminin-induced increase in expression of GCK and PKM2 glycolytic genes (Fig. 4b), consistent with the response observed when cells were transfected to overexpress FGFR5 (Fig. 3b). No differences were observed in CX36 transcript levels suggesting cell-to-cell connectivity and electrical coupling were not affected by laminin (Fig. 4c, left panel). Similarly, beta-cell culture on laminin did not affect the level of Integrin-alpha 6 transcript levels (Fig. 4c, right panel). Taken together, these data suggest that exposure to laminin induces upregulation of endogenous FGFR5 in beta-cell lines, as well as changes in expression of metabolic genes observed with FGFR5 overexpression, supporting a model of laminin-induced metabolic transformation via FGFR5 upregulation.

### Primary murine beta-cells cultured on laminin matrix exhibit increased FGFR5 protein expression and glucose-stimulated metabolism

**Figure 5:**
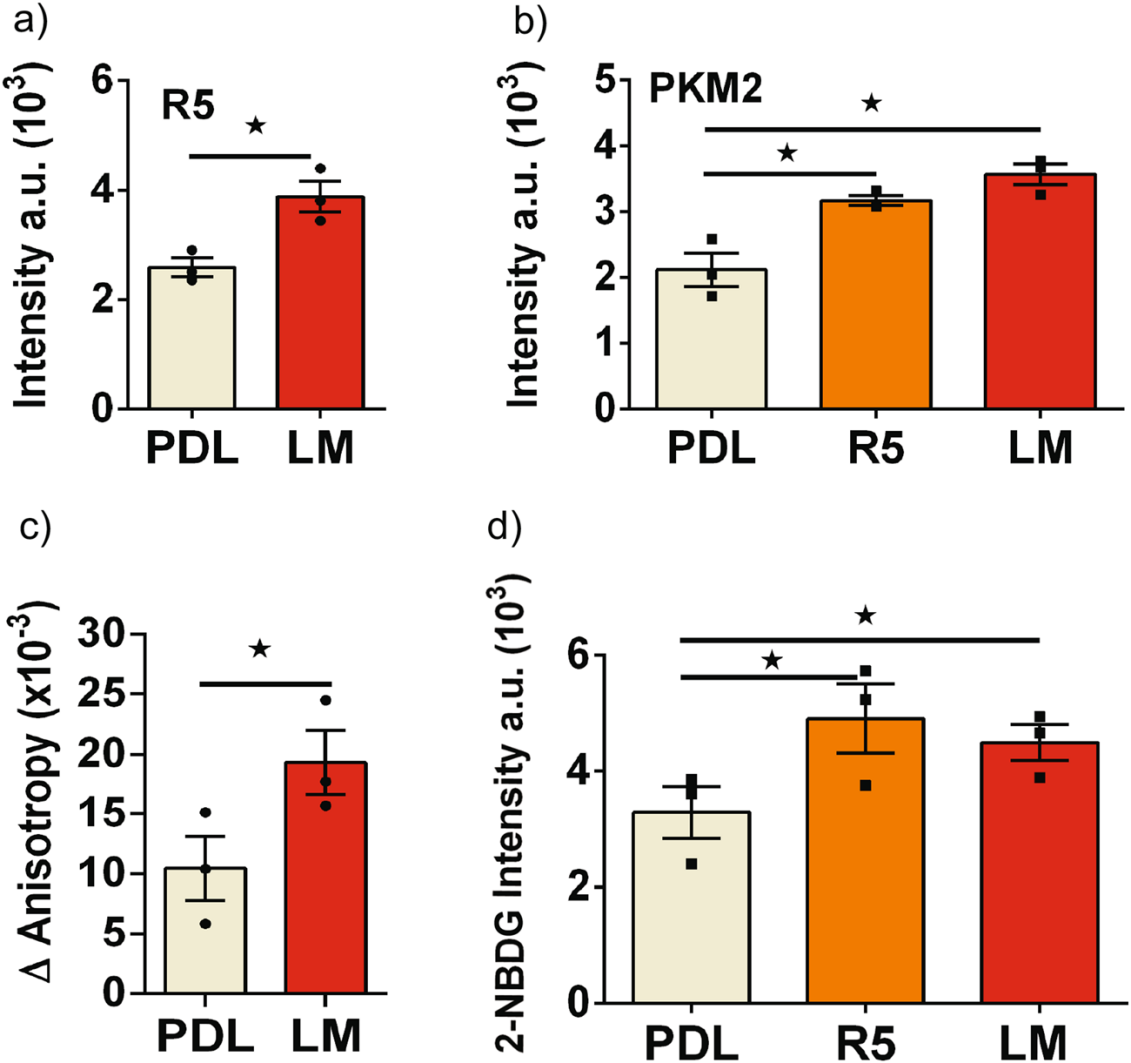
Primary murine islet beta-cells cultured on laminin matrix show increased levels of FGFR5 protein and glycolytic enzyme activity. To examine the effects of laminin induced transcript changes in a more physiologically relevant model, primary murine islets were dispersed on laminin matrix and the protein levels of previously tested genes were measured. Islets were dispersed onto poly-D-lysine (PDL) or laminin matrix (LM) in glass bottom dishes and transduced with mChe or R5 under a rat insulin promoter to identify beta-cells. (**a**) Endogenous FGFR5 immunofluorescence intensity of beta-cells. (**b**) Endogenous PKM2 immunofluorescence intensity of beta-cells plated on PDL or LM, or beta-cells overexpressing the R5 construct (R5) plated on PDL. (**c**) The Apollo-NADP^+^ response to glucose (1 to 15 mM) of beta-cells plated on PDL and LM reported as the change in anisotropy (ΔAnisotropy). (**d**) Dispersed beta-cells plated on PDL, plated on PDL and expressing the R5 construct (R5), or plated on LM were incubated with 15 mM glucose and 2 µM 2-NBDG for 20 min at 37 °C prior to imaging. Bars show the average indicated value ± SEM; symbols represent individual responses of n = 3 independent experiments. *, *p* < 0.05 using ANOVA and one-tailed t-test.

To translate from cell lines to primary cells, we measured responses in islets dispersed on laminin matrix using RIP driven-mCherry to specifically identify beta-cells (Fig. 5). Cells cultured on laminin showed a significant increase in endogenous FGFR5- and PKM2-associated immunofluorescence compared to cells cultured on PDL (Fig. 5a–b). A significant increase in FGFR5 protein expression was similarly confirmed in βTC3 cells (Fig. S6). Dispersed mouse beta-cells expressing R5 or plated on laminin also showed a decrease in the nuclear/cytoplasmic PKM2 ratio, consistent with cancer cell research indicating nuclear PKM2 promotes proliferation ^26^ (Fig. S7). To examine whether these protein level changes led to functional differences in glucose stimulated metabolism, we imaged glucose-stimulated responses of dispersed mouse beta-cells by expressing the Apollo-NADP^+^ sensor and RIP-driven mCherry (Fig. 5c). Beta-cells cultured on laminin showed a more robust 1 to 15 mM glucose-stimulated response compared to cells cultured on PDL. Finally, we measured 2-NBDG accumulation in beta-cells using RIP-driven mCherry co-expression as a surrogate for changes in GCK activity (Fig. 5d). 2-NBDG is a glucose mimetic that is transported into, and retained within, beta-cells by GCK-dependent phosphorylation. Beta-cells overexpressing FGFR5 or plated on laminin showed significantly greater 2-NBDG-associated fluorescence than control cells, consistent with an increase in GCK activity. Taken together, these data suggest laminin-induced FGFR5 expression affects beta-cell metabolism in primary beta-cells similar to the R5 response in beta-cell lines.

### Overexpression of truncated FGFR5 blocks laminin-induced metabolism in beta-cells

**Figure 6:**
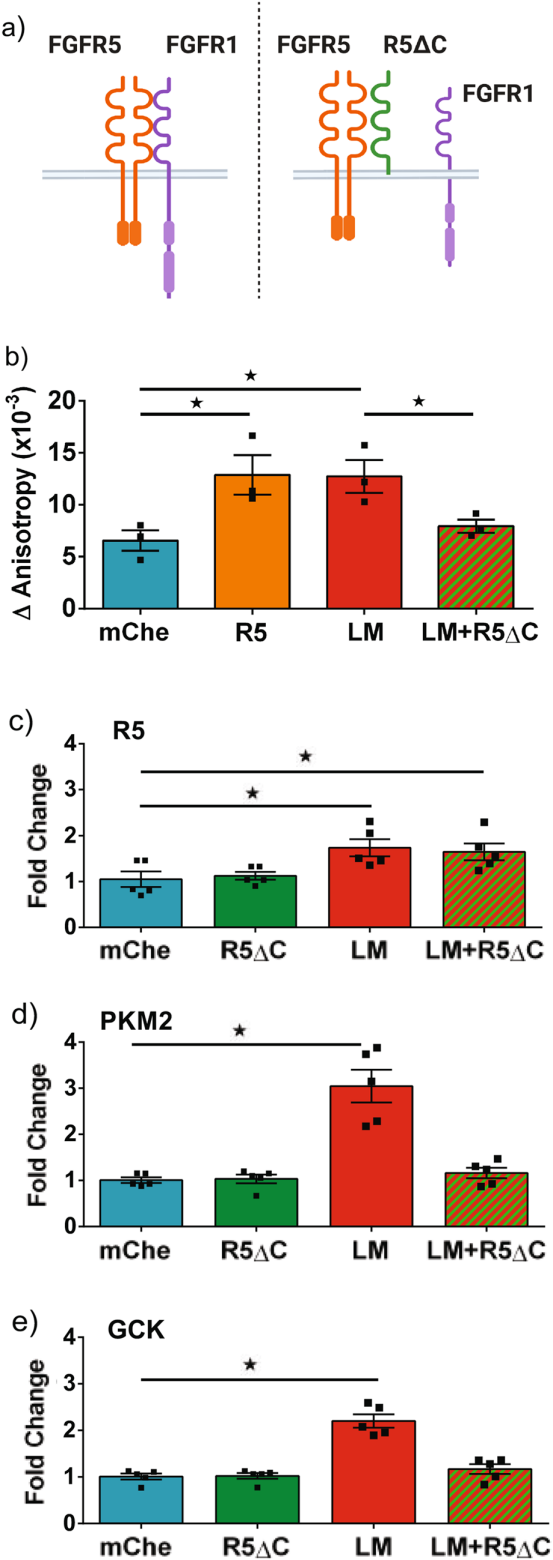
R5ΔC expression blocks laminin-induced metabolism. To examine the role of FGFR1 in the FGFR5 induced metabolic remodeling, R5ΔC was overexpressed in beta-cells plated on laminin matrix to disrupt the FGFR5/FGFR1 signaling complex. (**a**) FGFR5 binds with FGFR1 to create a 2:1 FGFR5:FGFR1 signaling complex. This signaling complex can be disrupted by overexpression of R5ΔC, which outcompetes FGFR1 for binding in the heterocomplex as illustrated. The 2:1 FGFR5/R5ΔC complex cannot signal and therefore neutralizes FGFR5 regulation of FGFR1 signaling. (**b**) Dispersed mouse beta-cells were transduced with adenovirus to co-express Venus-Apollo-NADP^+^ (CMV promoter) and mCherry (mChe), R5-mCherry (R5), or R5ΔC (all RIP promoter) and cultured on either PDL (mChe, R5) or laminin (LM, LM + R5ΔC). The Apollo-NADP^+^ response to 1 mM and 15 mM glucose (ΔAnisotropy) was collected from the mCherry-positive beta-cells as determined by RIP promoter driven expression. (**c**–**e**) βTC3 cells cultured on PDL (mChe, R5ΔC) or laminin (LM, LM + R5ΔC) were transduced with adenovirus to express mCherry (mChe and LM) or R5ΔC (RIP promoter) prior to comparing transcript expression by qPCR. Bars show the average indicated value ± SEM; symbols represent individual responses of n = 3 (**b**) and 5 (**c**,**d**,**e**) independent experiments. *, *p* < 0.05 using ANOVA followed by one-tailed t-test.

To determine whether laminin regulates beta-cell responses through FGFR5, we measured the impact of truncated receptor (R5ΔC) expression on laminin-induced responses (Fig. 6). We previously showed that R5ΔC competes with FGFR1 for binding to the 2:1 FGFR5:FGFR1 complex to effectively block FGFR5 activity^16^ (Fig. 6a). We first measured the impact of R5ΔC on glucose-stimulated NADPH-response in dispersed mouse islet beta-cells (Fig. 6b). These data show the laminin-enhanced NADPH response is blocked by R5ΔC, consistent with laminin having effect through FGFR5. We subsequently measured the impact of R5ΔC on FGFR5, PKM2, and GCK transcript expression in βTC3 cell lines (Fig. 6c–e). These data show R5ΔC alone had no effect on the level of each of these transcripts and did not block laminin-induced expression of FGFR5. In contrast, R5ΔC did block the laminin-induced expression of PKM2 and GCK. Overall, these data suggest that laminin upregulates beta-cell FGFR5 to subsequently remodel metabolism.

### FGFR5 overexpression in human beta-cells induces a more dynamic glucose stimulated NADPH response

**Figure 7:**
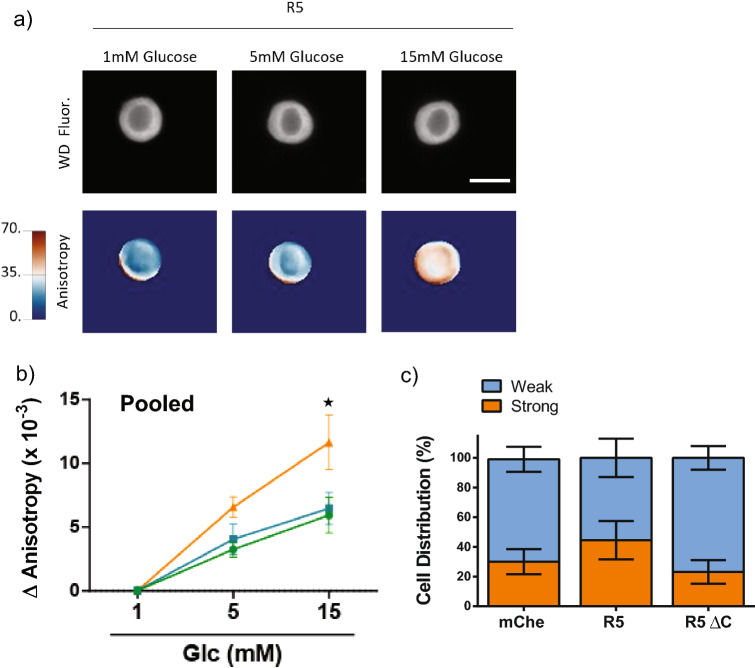
FGFR5 overexpression in primary human beta-cells induces a more robust glucose stimulated NADPH response​. To further support the physiological relevancy of our findings, R5 overexpression was examined in primary human islet samples. Human islets were dispersed to single cells on PDL treated glass bottom dishes and co-transduced with Apollo-NADP^+^ sensor (CMV promoter) and either mChe, R5, or R5ΔC (RIP promoter). (**a**) Widefield fluorescence (WD Fluor) and anisotropy images from a representative beta-cell at multiple glucose concentrations, as indicated. (Scale bar = 5 μm; anisotropy lookup table for widefield anisotropy =  × 10^–2^). (**b**) Apollo-NADP^+^ response (Anisotropy) of mCherry positive beta-cell cells overexpressing R5, mChe, and R5ΔC in response to sequential glucose concentrations, as indicated. (**c**) The Apollo-NADP^+^ responses of mChe, R5 and R5ΔC expressing beta-cells (traces shown in Fig. S9) were classified as either strong (orange) or weak (dark blue) glucose responders based on the mean mChe cell response. Bars indicate % cell distribution for each construct and represent the average ± SEM of n = 7 independent experiments. *, *p* < 0.05 using ANOVA followed by one-tailed t-test.

To measure the impact of FGFR5 on human beta-cell metabolism, we expressed mChe, R5, and R5ΔC in dispersed human beta-cells using RIP-driven adenovirus and imaged the glucose-stimulated response using Apollo-NADP^+^ (Fig. 7a). Tissue from donors of varying age, sex, and BMI consistently showed R5-expression increased the glucose-stimulated response (Fig. S8a–g) resulting in a statistically significant response compared to mChe and R5ΔC expressing beta-cells (Fig. 7b). Additionally, the compiled single-cell metabolic traces showed a trend to increasing the fraction of strong responders similar to mouse beta-cell data (Fig. 7c; Fig. S9). Overall, these data show that increased expression of FGFR5 enhances glucose-stimulated metabolism in human beta-cells.

## Discussion

**Figure 8:**
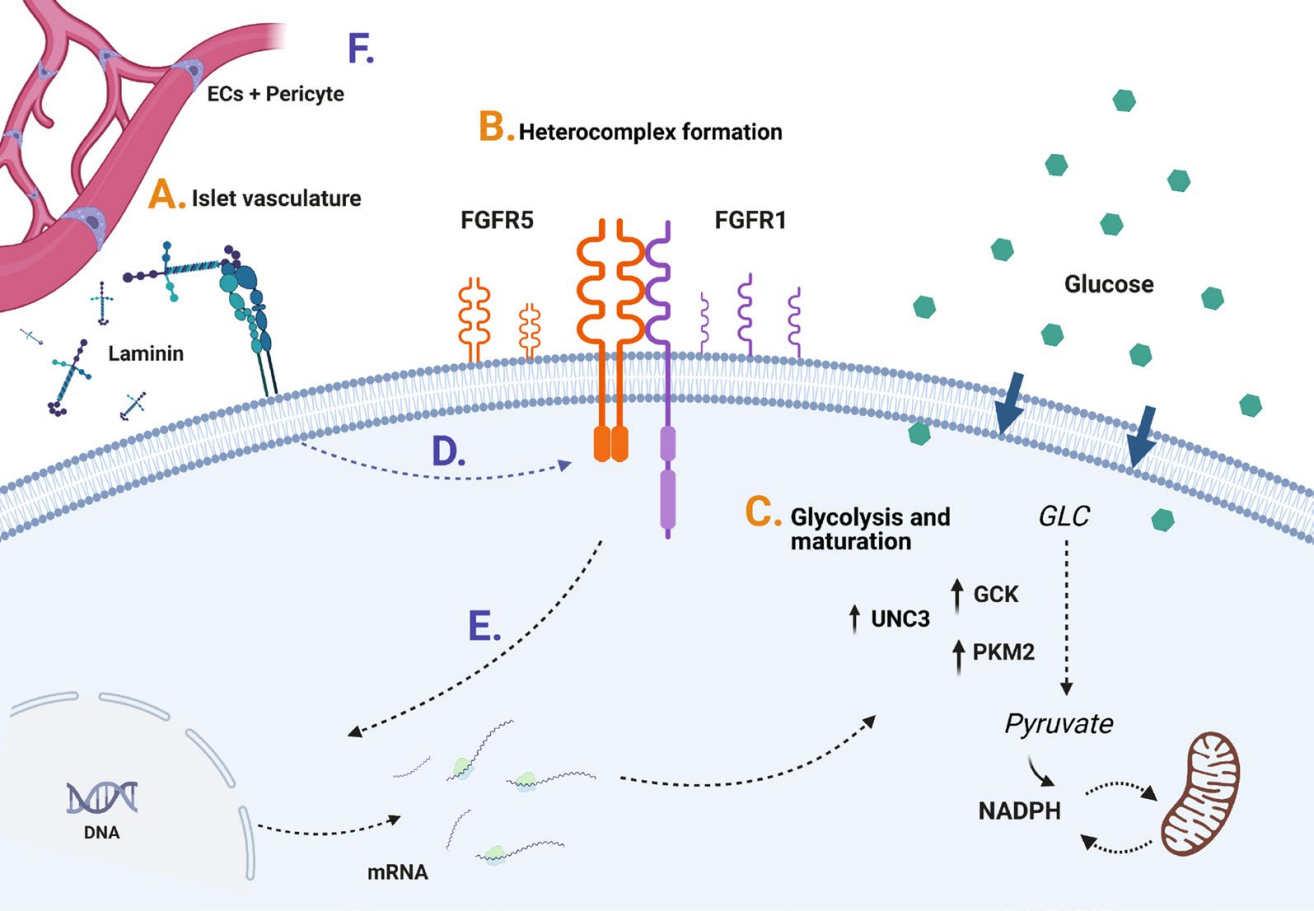
Model of laminin-induced FGFR5 expression leading to enhanced beta-cell glucose metabolism. (**A**) Islet vasculature: Endothelial cells (ECs) and pericytes form the islet vasculature, and deposit and remodel islet basement membrane. Varied localization and/or phenotype of EC/pericytes establishes the beta-cell microenvironment. Our data show upregulation of beta-cell FGFR5 induced by binding to laminin matrix. (**B**) Heterocomplex formation: Upregulation of FGFR5 protein at the cell membrane results in formation of a 2:1 heterocomplex of FGFR5:FGFR1 previously shown to depend on the C-terminus of FGFR5^16^. (**C**) Glycolysis and maturation: FGFR5/FGFR1 signaling induces expression of the functional maturity marker UCN3 as well as rate-limiting enzymes of glycolysis (PKM2, GCK). (**D**) Multiple studies done by our lab have now highlighted the role of ECM in regulating FGFR expression. Future studies will focus on examining the mechanism underlying this signaling pathway. (E) To develop this model in greater detail, future studies will also look at the signaling pathways underlying the intracellular transcriptional and metabolic changes we have observed with FGFR5 expression. (F) Lastly, more work must be done to understand how changes in the microenvironment impact beta-cells. This includes how ECM remodeling by ECs and pericytes affects beta-cell metabolism and maturity and the specific mechanisms of FGFR-regulated intracellular signaling. (Made in ©BioRender—biorender.com).

By imaging the Apollo-NADP^+^ sensor, we showed that FGFR5 expression positively impacts glucose-stimulated metabolism in living beta-cell lines and mouse and human beta-cells. This enhanced response to glucose was associated with increased expression of key rate-limiting enzymes of glycolysis (GCK, PKM2) and functional maturity markers (MAFA, UCN3). Additionally, beta-cells plated on laminin exhibited increased FGFR5 expression compared to cells plated on PDL, with concomitant increases in glucose stimulated NADPH metabolism, PKM2 expression, and GCK activity. Overall and in the context of our previous work, these data suggest that beta-cells upregulate FGFR5 expression in the presence of a laminin microenvironment resulting in interaction with FGFR1 to enhance beta-cell glucose metabolism (Fig. 8A–C). Future studies will need to explore the intracellular signaling from integrin activation that induces FGFR5 expression (Fig. 8D), and FGFR5/FGFR1 signaling that drives changes in metabolism (Fig. 8E). It is of additional interest to explore the role of islet ECs and pericytes in modifying laminin deposition and discern in the context of FGFR5 whether this pathway contributes to transcriptional and/or functional changes within islets (Fig. 8F).

We previously showed basement membrane ECM, and in particular laminin, modulates beta-cell expression of endogenous FGFR1 receptor activity through α_6_β_1_ integrin activity^14^. These data are consistent with other studies showing laminin spurs beta-cell maturation and a more functional state^19^. Our data in this study reveal increased FGFR5 expression in beta-cells cultured on laminin thereby suggesting an overall increase in FGFR5:FGFR1 ratio (Fig. 8). Originally identified by strong expression in the pancreas, kidney, brain, and lung, FGFR5 is deficient for the split tyrosine kinase domain present in canonical FGFRs required to initiate intracellular signaling cascades, suggesting a unique role as a coreceptor rather than an independent signaling activator^27,28^. FGFR5 expression has generally been associated with inhibition of cellular proliferation, differentiation, and increased cell adhesion^17^ but has also been defined as a co-receptor for FGFR1 through formation of a 2:1 membrane heterocomplex that responds to FGF2 with enhanced ERK1/2 protein activation^16,18^. A recent study of maturity markers as an indicator of beta-cell heterogeneity demonstrated that older beta-cells (> 1 year) exhibit an increased FGFR5: FGFR1 transcript ratio^3^. Thus, our data is consistent with cells increasing FGFR5 in the presence of laminin to limit unbound FGFR1 expression, resulting in a more mature beta-cell phenotype. We therefore propose FGFR5 and the resulting FGFR5:FGFR1 heterocomplex act as an intermediary for outside-in signaling between laminin matrix and beta-cell metabolic maturity.

The significant transcriptional and functional heterogeneity of beta-cells within islets has been categorized based on a variety of parameters that includes islet architecture. Specifically, the varied microenvironment due to heterogenous interaction with the capillary network could provide unique influence on beta-cell phenotype^29,30^. Recent studies have shown that 3D islet architecture working in conjunction with planar cell polarity molecules such as *Flattop* can influence beta-cell function and maturity^2^. The majority of ECM within islets is deposited by the ECs and pericytes, thus establishing a diverse beta-cell microenvironment^6,31^. Pericytes, recently proposed to be the primary source of islet laminins, surround the microvasculature in islets with a ~ 0.5:1 pericyte-to-EC ratio that contributes to controlling islet blood flow and decreases with progression of diabetes^6,32^. In this context, beta-cells could therefore be variably exposed to laminin based on proximity to ECs/pericytes and/or variation in EC/pericyte phenotype^33^.

Considering a role for islet architecture in regulating FGFR5 expression and activity, it is informative to consider when basement membrane in islets could be dynamically remodeled by changes in ECs/pericytes. Examples include (a) angiogenesis-associated ECM remodeling involving cycles of pericyte detachment, basement membrane degradation, sprouting and pericyte reattachment, and vessel maturation that could regulate local beta-cell FGFR5 activity from low (pericyte detachment and basement membrane degradation) to high (pericyte reattachment and vessel maturation), (b) endothelial-to-mesenchymal transition (EndoMT) associated with several pathological conditions including senescence that involve ECM remodeling including varied laminin deposition^34^, and (c) changes in vessel phenotype (e.g., capillary to post-capillary) resulting in changes in laminin composition^33^ and pericyte coverage. Thus, several well-established changes in EC/pericyte phenotype could dynamically impact FGFR5 expression and activity in neighboring beta-cells. Overall, the microenvironment established by varied laminin deposition could regulate FGFR5 function and be dynamically modified by physiologically relevant switches in EC/pericyte phenotype.

Insulin resistance is a hallmark of type 2 diabetes that early in the disease is compensated for by increased beta-cell insulin secretion^35^. This compensation eventually becomes inadequate due to beta-cell dysfunction in a proportion of patients who then progress into full blown diabetes. Islet inflammation including cytokines (IL-1, IL-6, and TNF-alpha), immune cells, and fibrosis have been implicated as both markers and drivers of beta-cell dysfunction^36,37^. Notably, we previously showed cytokine stimulation increases beta-cell FGFR5 expression and enhances cell survival^16^. Therefore, we propose that FGFR5 could play a role in the early compensatory response to insulin resistance. In this model, modified laminin secretion by inflamed ECs/pericytes^33^ would trigger beta-cell FGFR5 expression to enhance metabolism and survival (Fig. 8), both of which would impede the development of type 2 diabetes.

## Materials and methods

### Cell culture

Cell lines used in this study were cultured in cell media at 37 °C in humidified 5% CO_2_. βTC3 cells were cultured in DMEM containing 4.5 g/L glucose, 15% (vol/vol) horse serum, 2.5% (vol/vol) FBS, and 5% (vol/vol) penicillin/streptomycin (P/S). INS1-E cells were cultured in RPMI media containing 4.5 g/L glucose, 5% (vol/vol) FBS, 1.19 g/L HEPES, 1.748 µl/L 2-Mercaptoethanol and 5% (vol/vol) P/S.

### Construct expression

βTC3 and INS1-E cells were plated on 35-mm No. 1.5 glass bottom dishes (MatTek Corp.) for fluorescence imaging experiments. At approximately 50–60% culture confluence, the cells were transfected using PolyJet (SigmaGen Laboratories; 3 μl Polyjet:1 μg cDNA in culture media) with relevant plasmid constructs, including wild-type FGFR5 exhibiting a mCherry fluorescent tag (R5), FGFR5 deficient in the intracellular domain exhibiting a mCherry fluorescent tag (R5ΔC), or mCherry monomer control plasmid (mChe) (as indicated)^18^. Transfection media was replaced with fresh culture media 16 h post-transfection and samples were imaged 72 h post-transfection. All live cell imaging experiments were conducted using imaging media (125 mM NaCl, 5.7 mM KCl, 2.5 mM CaCl_2_, 1.2 mM MgCl_2_, 10 mM HEPES, and 2 mM glucose; pH 7.4). For βTC3 qPCR analysis and dispersed mouse and human islet analyses, we used previously constructed adenovirus (24 h at 1:10 dilution or ~ 2 × 10^7^ IFU/ml) that drives expression of mCherry, R5-mCherry, and R5ΔC-mCherry in beta-cells using a rat insulin promoter^16^. Viral titers were measured using the Adeno-X rapid titer kit (Clontech) following the manufacturer’s protocol.

### Western immunoblotting

Cell cultures were washed with sterile PBS and harvested with addition of cell lysate buffer on ice. Whole cell lysate protein concentration was determined by colorimetric protein assay (BioRad) using BSA as a standard. The equivalent of 10 μg total protein per lane was separated by 10% SDS-PAGE and transferred to nitrocellulose membranes for non-specific blocking (5% milk/TBS-T; 1 h; RT) and overnight incubation (4 °C) with the following antibodies diluted in blocking solution: mCherry (E5D8F) rabbit monoclonal (1:500; CST) and GAPDH (1:1000; SCBT). Blots were subsequently incubated with horseradish peroxidase-linked anti-rabbit and anti-mouse secondary antibodies (1:2000; CST; 45 min; RT) before detection by enhanced chemiluminescence.

### Anisotropy imaging of Apollo-NADP^+^

Apollo-NADP^+^ is a genetically encoded family of single-color sensors that respond to changes in cytoplasmic NADPH/NADP^+^ redox state with changes in steady-state fluorescence anisotropy^20^. Imaging mVenus Apollo-NADP^+^ in low-throughput experiments was performed using a LSM710 confocal microscope (Zeiss) equipped with a Chameleon two-photon laser (Coherent)^20^. Cells were imaged using a 63 × /1.4 NA oil-immersion objective, 25.2 μs pixel dwell time, image size of 512 × 512 pixels, detector gain of 700, and 7% two-photon (2P) laser at 950 nm. Anisotropy images, based on the parallel (I_||_) and perpendicular (${\text{I}}_{ \bot }$) intensities, were simultaneously collected using the external non-descanned binary GaAsP (BiG) detector equipped with an infrared light–blocked emission bandpass filter (500–550 nm, Chroma), polarizing beam splitter (Edmund Optics), and clean-up polarizers (Chroma)^15^. Cerulean and mVenus Apollo-NADP^+^ sensors were also imaged on a custom built Applied Scientific Instrumentation (ASI) widefield microscope system to achieve higher throughput^38^. Anisotropy images were collected using a 40 × /0.75 NA air objective lens using 405 nm (LED Engin), 505 nm (LumiLEDs), and 590 nm (Roithner Lasertechnik) excitation of Cerulean, mVenus, and mCherry constructs, *respectively*. An Iris 15 Scientific CMOS (sCMOS) camera was used to capture images. The system also includes an ECFP/EYFP/mCherry bandpass filter set (Chroma), a polarizing beam splitter (ASI c-60 D cube), a clean-up polarizer (Chroma), and excitation filter (Chroma). The two-photon and widefield I_||_ and ${\text{I}}_{ \bot }$ images were background corrected using a rolling ball filter prior to calculation of the anisotropy image (Anisotropy = (${\text{I}}_{||} - {\text{I}}_{ \bot }$) / (${\text{I}}_{||} + 2{\text{I}}_{ \bot }$) including g-factor and high NA correction using a custom ImageJ (NIH) script. One ROI/cell was made in the cytoplasmic region to exclude the nucleus using ImageJ.

### Pancreatic islets

The procedure for murine islet isolation has been approved by the Animal Care Committee of the University Health Network, Toronto, Ontario, Canada, in accordance with the policies and guidelines of the Canadian Council on Animal Care (Animal Use Protocol number 1531) and following ARRIVE guidelines. Islets were harvested from C57BL6 male mice using a collagenase-p (Roche Applied Science) based digestion protocol. Experimental mice ranged in age from 2 to 9 months. Following isolation, islets were equilibrated in RPMI 1640 media containing 11 mM glucose, 10% (vol/vol) FBS, 1.19 g HEPES and 5 units/ml P/S. Isolated islets were transferred into an Eppendorf tube (1.5 ml) containing 50 µl RPMI islet media and 50 µl Trypsin:EDTA solution (see “Cell culture” methods). Each tube was immersed in a water bath at 37 °C and shaken gently by hand for 12 min. Once fully dissociated, the islet slurry was supplemented up to 1 ml total volume with RPMI islet media. Glass bottom plates (48-well) were incubated with a layer of Poly-D-Lysine (PDL) hydrobromide (Sigma-Aldrich, Product #P6407) for 1 h at 37 °C to promote dispersed islet cell adhesion. Other experiments included incubation with the ECM protein laminin (Sigma-Aldrich, Product #L2020) for 30 min at 37 °C post-PDL coating. Islet slurry was added to each well of a 48-well glass bottom plate (330 µl/well) coated with PDL. Experimental treatments were added, as indicated. Human islets for research were provided by the Alberta Diabetes Institute IsletCore at the University of Alberta in Edmonton (www.bcell.org/adi-isletcore) with the assistance of the Human Organ Procurement and Exchange (HOPE) program, Trillium Gift of Life Network (TGLN), and other Canadian organ procurement organizations. Islet isolation was approved by the Human Research Ethics Board at the University of Alberta (Pro00013094). All donor families gave informed consent for the use of pancreatic tissue in research. All methods were performed in accordance with the relevant guidelines and regulations. Identification of islet preparations are as follows: sample 1- R356, sample 2- R382, sample 3- R392, sample 4- R411, sample 5- R412, sample 6- R417, sample 7- R418. Donor information can be found in the ADI IsletCore donor and sample database.

### qPCR

RNA extracted from βTC3 cells was purified using RNeasy Mini Kits (Qiagen). cDNA libraries were subsequently generated using the High-Capacity cDNA Reverse Transcription Kit (Applied Biosystems). The NCBI Primer BLAST tool (NIH) was used to design relevant oligonucleotide primers (detailed information can be found in Supplementary Table S1). GAPDH was selected as the control housekeeping gene. qPCR cycling was conducted using a LightCycler® 480 Instrument II (Roche Life Sciences) and performed using the ΔΔCt method of analysis, which compares the CT values of a housekeeping gene (GAPDH) to each gene of interest in experimental and control conditions.

### Mitochondrial membrane potential (MMP)

Cells were pre-incubated with imaging media supplemented with 1 mM glucose and 5 µM Rh123 (7 min at 37 °C) to achieve dye self-quenching. After replacing media, cells were placed in the stage top incubator (37 °C) of the ASI widefield microscope for imaging. A 15 mM glucose bolus was added 5 min prior to imaging treated samples. Fluorescence samples were excited using a 405 nm LED and collected through an EYFP bandpass filter.

### Immunofluorescence

Cell lines plated on glass bottom dishes (35 mm; MatTek Corp.) and dispersed islets plated on glass bottom plates (48 well; MatTek Corp.) were cultured on laminin and/or transduced with viral constructs for 24 h. Once fixed with 100% ice-cold methanol (-20 °C; 15 min), samples were blocked for 1 h in 5% goat serum/PBS at RT. Samples were consequently incubated for 24 h (4 °C) with FGFR5 antibody (Invitrogen; #PA5-21,516) diluted 1:1000 in 1.5% goat serum/PBS, or PKM2 antibody (Cell Signalling Technology; #3198) diluted at 1:500 in 1.5% goat serum/PBS. The antibodies were subsequently detected using anti-rabbit Alexa Fluor 560 diluted at 1:1000 in block solution (5% goat serum/PBS; RT for 45 min). Nuclei were counterstained for 3 min at RT with DAPI (5 µg/ml). All samples were imaged using the 63 × /1.4–NA oil-immersion objective lens of the ASI wide field microscope.

### Imaging 2-NBDG (2-(*N*-(7-Nitrobenz-2-oxa-1,3-diazol-4-yl)Amino)-2-Deoxyglucose) accumulation

2-NBDG is a fluorescent glucose mimetic that is retained in cells once phosphorylated by a hexokinase. Measuring 2-NBDG fluorescence accumulation in beta-cells allows insight into the level of GCK activity. Cells were incubated in imaging media supplemented with 15 mM glucose for 30 min (37 °C) prior to imaging. Fresh media was exchanged containing 15 mM glucose and 2 µM 2-NBDG, and samples were incubated for 20 min at 37 °C before being imaged with the ASI widefield microscope. Fluorescence images were excited using a 405 nm LED and collected through an EYFP bandpass filter.

### Insulin assay

βTC3 cells plated in six well culture dishes were transfected with the mChe and R5 constructs, as indicated. After reaching ~ 80% confluency at 48 h post transfection, the cells were equilibrated with 1 mM glucose in imaging media for 30 min to achieve baseline stimulation. The cells were sequentially stimulated with 1 mM and 15 mM glucose, with culture media being collected after each 1 h incubation at 37 °C. The cells were harvested using Trypsin:EDTA (37 °C for 5 min) and counted manually using a hemocytometer (100 µL cell lysate mixed with 100 µL Trypan Blue dye). Harvested cells were centrifuged and resuspended in acid ethanol (3.75 ml EtOH, 75 µL 1 M HCl, 1.175 ml dH_2_O, 5 µL Triton X-100) to liberate intracellular insulin (total insulin). The media and lysate samples were diluted 10 × and 1000x, *respectively,* prior to assay using an Insulin ELISA kit (Millipore). Total insulin was normalised to cell count and secreted insulin was normalized to total insulin.

### Anisotropy image presentation

A threshold and median filter were applied to anisotropy images before display using a vik look-up table. In anisotropy imaging, background regions are inherently noisy and distract unnecessarily from the cell anisotropy. Thus, we followed common practice in setting background regions that are below a threshold fluorescence intensity to zero anisotropy.

### Data and resource availability

Data supporting the findings of this study are available upon reasonable request.

### Statistics

Results were analysed for significance using unpaired two-sample one-tailed t-tests as well as one-way analysis of variance (ANOVA). Statistical analysis was performed using the Microsoft Excel data analysis pack and GraphPad Prism 6.0. Graphical error bars represent ± standard error of the mean (SEM) or standard deviation (SD) for relevant analyses, as indicated. Experiments were performed in replicate 3–5 separate times. Live cell experiments contained approximately 15–35 cells per run. Human islet data are shown as individual samples (mean ± SD) and pooled responses (mean ± SEM).

## Supplementary Information

Supplementary Information.

## Data Availability

The datasets generated during and/or analysed during the current study are available from the corresponding author on reasonable request.
