# Supplementary material for: Laminin matrix regulates beta-cell FGFR5 expression to enhance glucose-stimulated metabolism
Source: Sci Rep. 2022 Apr 12;12:6110. doi: 10.1038/s41598-022-09804-7 (PMC9005713; doi:10.1038/s41598-022-09804-7)
Supplement: Supplementary file 1 — Supplementary Information. [file 41598_2022_9804_MOESM1_ESM.pdf]

S1)

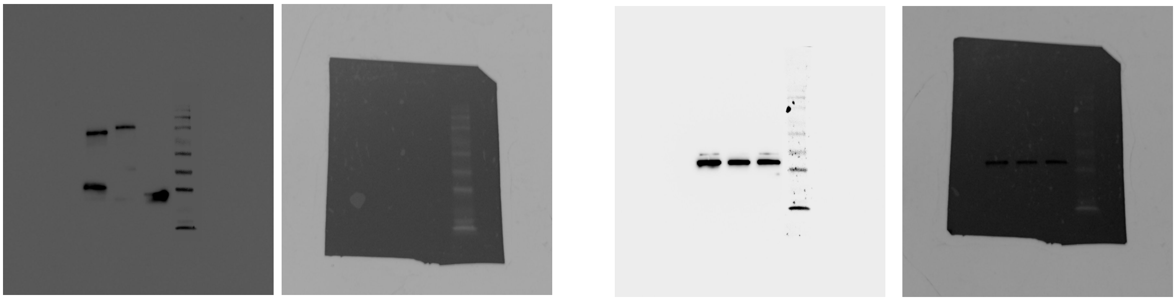

Uncropped Ab: mChe | Ab:mChe Live | Uncropped Ab: GAPDH | Ab: GAPDH Live

Figure S1. Uncropped images of Western blot.

Left image is enhanced chemiluminescence (ECL) right images are white light. Size marker was Bio-Helix PMB13-0500 run on SDS -PAGE .

S2)

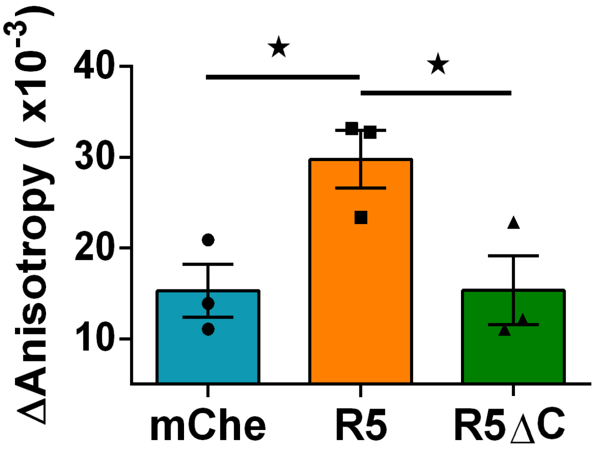

Figure S2. Comparison of glucose-stimulated changes in anisotropy (ΔAnisotropy). INS1E cells expressing the Apollo-NADP$^+$ sensor and either mCherry (mChe), mCherry-tagged FGFR5 (R5), or mCherry-tagged C-terminal truncated FGFR5 (R5ΔC) were exposed to 1 mM and 15 mM glucose, followed by 2 mM diamide to deplete NADPH (sensor anisotropy reduced to baseline). This figure alternatively plots the data shown in Figure 1d based on changes in anisotropy (ΔAnisotropy) between 1 mM and 15 mM glucose stimulation. Each symbol represents the average of cell replicates (>10 cells) for an independent experiment. Bars represent the average ± SEM of n = 3 independent experiments. *, $p < 0.05$ using ANOVA followed by one-tailed t-test.

S3)

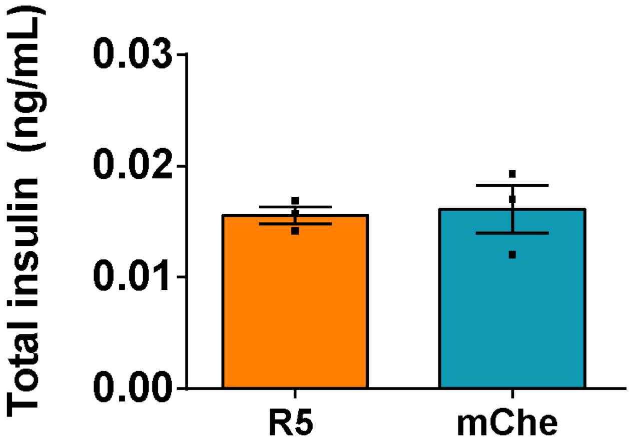

Figure S3. R5 expressing cells exhibit no change in total insulin response. βTC3 cells overexpressing R5 were lysed with acid ethanol solution to collect total insulin. Insulin levels were detected using a commercial ELISA. Insulin secretion levels (Fig. 1g) were normalized to the total insulin measurement.

S4)

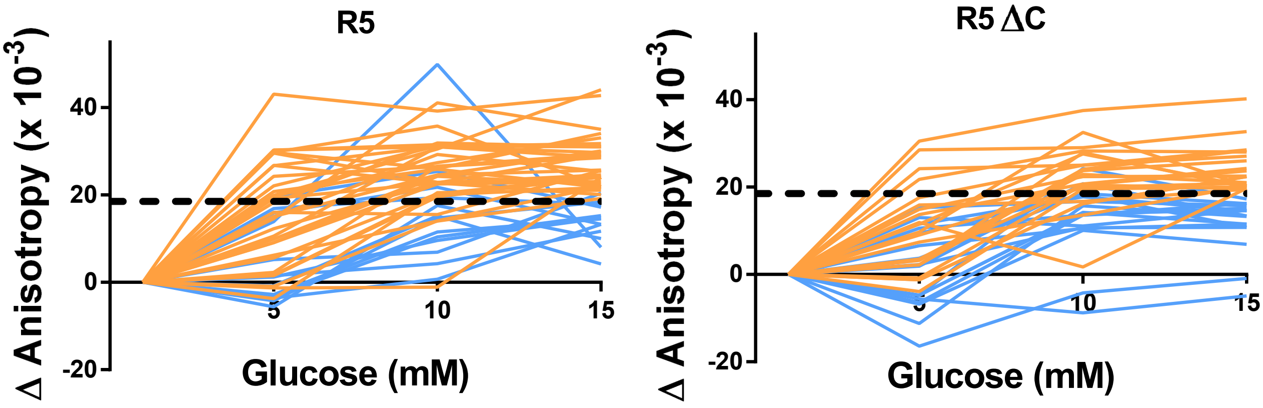

Figure S4. Individual glucose-stimulated NADPH responses of R5- and R5ΔC-expressing mouse beta-cells. Mouse islets were dispersed to single cells onto glass bottom dishes and co-transduced with the Apollo-$NADP^+$ sensor expressing a CMV promoter and one of the mCherry constructs (mChe, R5, or R5ΔC) expressing a rat insulin promoter. Single cell traces from dispersed beta-cells expressing R5 and R5ΔC are grouped into strong (blue) and weak (orange) response categories based on a threshold determined by the mCherry expressing control cell population (shown in Fig. 2c).

S5)

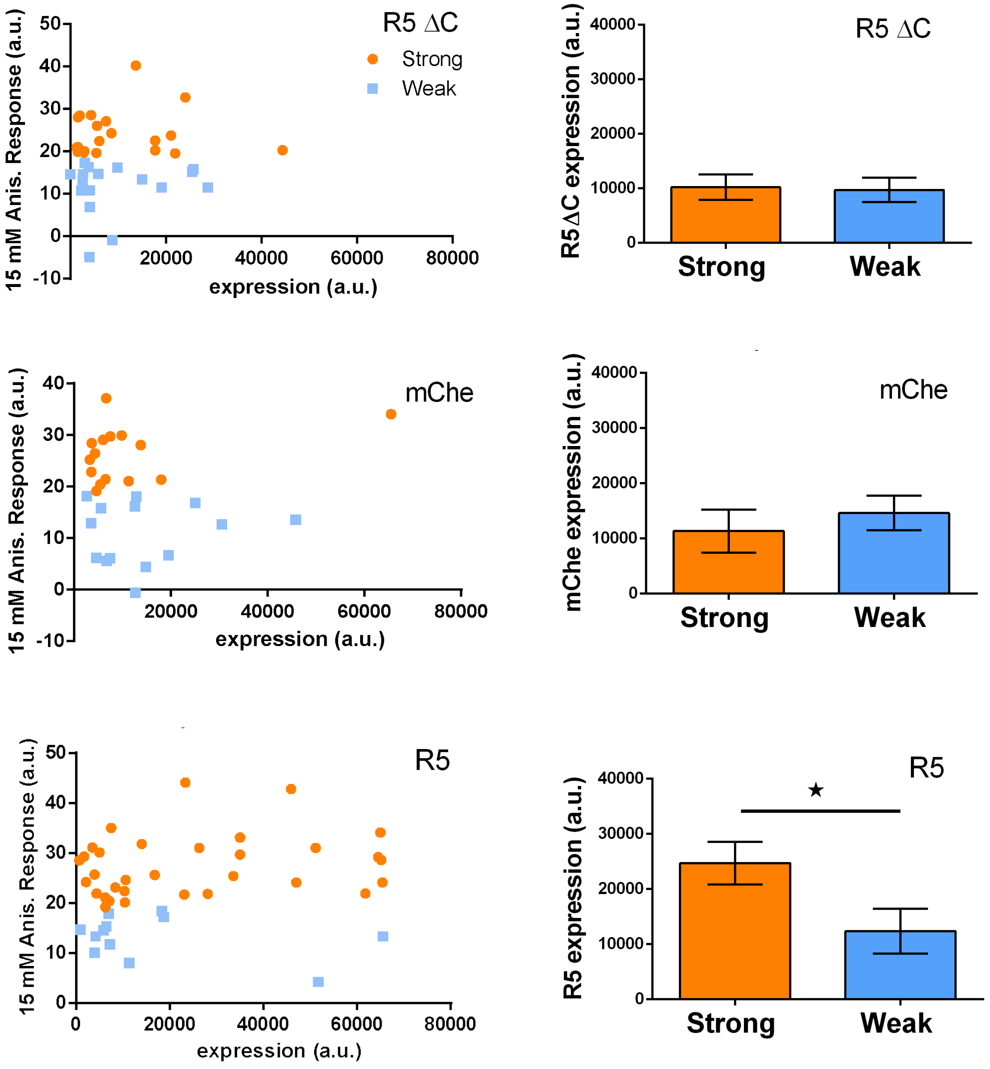

Figure S5. Beta-cells with greater R5 expression exhibit stronger metabolic responses. Increasing R5 expression levels in mouse beta cells are correlated with stronger metabolic dynamics compared to R5ΔC- and mCherry-expressing cells as determined by Apollo $NADP^+$ glucose dose response analysis (single cell tracking data from Fig.2 further analyzed). Individual cell responses show R5 construct expression plotted against the 15 mM anisotropy response (left panels) as well as the averaged construct expression according to the strong (orange) and weak (blue) response delineation shown in Fig.2 (right panels). Bars show the average indicated value ± SEM; dots represent individual responses of dispersed beta cells. *, $p < 0.05$ using ANOVA followed by one-tailed t test.

S6)

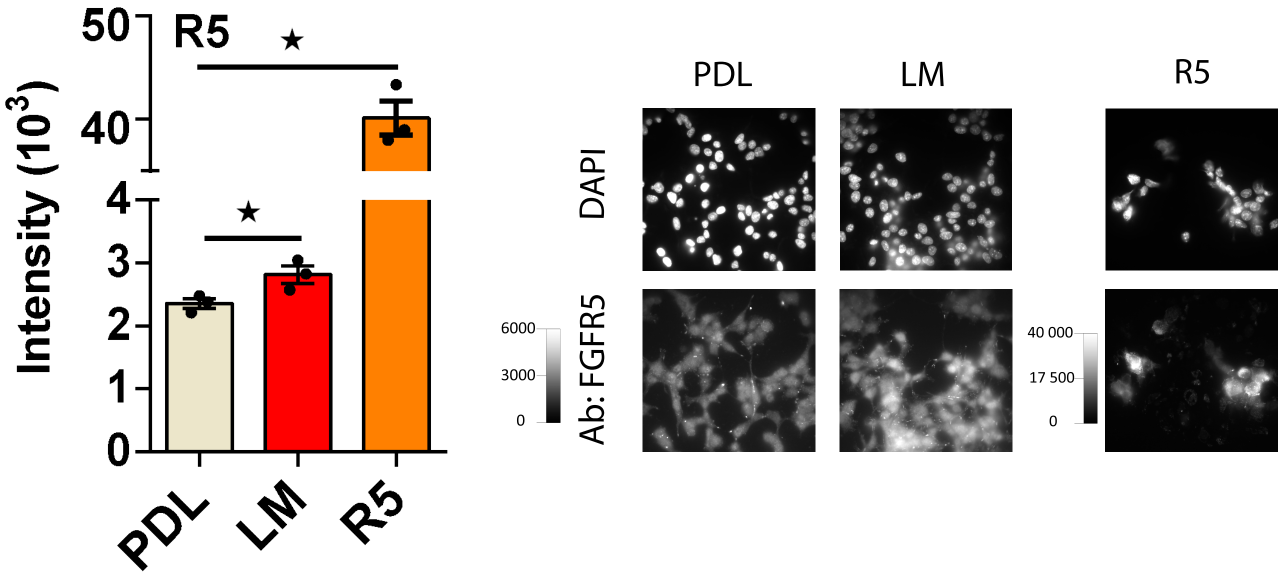

Figure S6. Immunofluorescent detection of endogenous FGFR5 protein in βTC3 cells. βTC3 cells were plated on glass bottom dishes coated with poly-D-lysine (PDL), laminin (LM), and on PDL overexpressing R5. Samples were immunostained for FGFR5 expression levels. The presented images have processed using a gaussian blur (sigma radius 2). The PDL and LM lookup table has been corrected so the minimum and maximum displayed values are 0-6000. For presentation purposes, the minimum and maximum displayed values by the R5 lookup table are set to 0- 40 000 to accommodate for increased intensity. Data are plotted as the mean ± SEM; dots represent individual responses of n = 3 separate experiments. *, $p < 0.05$ by one-tailed t-test.

S7)

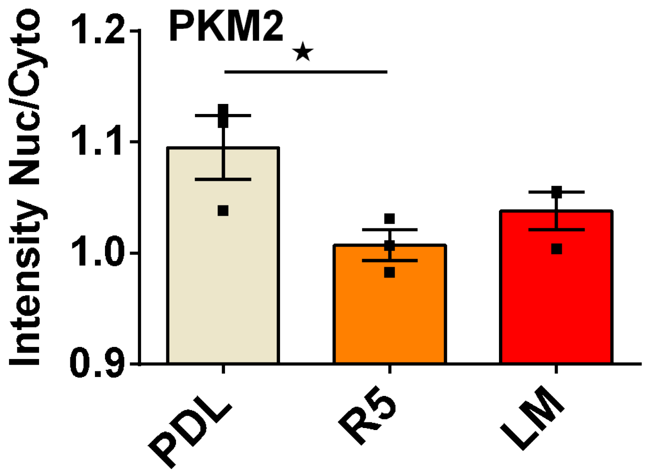

Figure S7. PKM2 localization to the nucleus of dispersed mouse beta-cells. Mouse islets were dispersed onto poly-D-lysine (PDL) and laminin matrix (LM) in glass bottom dishes and transduced with mCherry or R5 under a rat insulin promoter to identify beta-cells. Endogenous PKM2 expression was identified by immunofluorescence detection and the nuclear/cytoplasmic ratio determined using DAPI stain (analysis of data shown in Fig. 5b). Data are plotted as the mean ± SEM; dots represent individual responses of n = 3 separate experiments. *, $p < 0.05$ by one-tailed t-test.

S8)

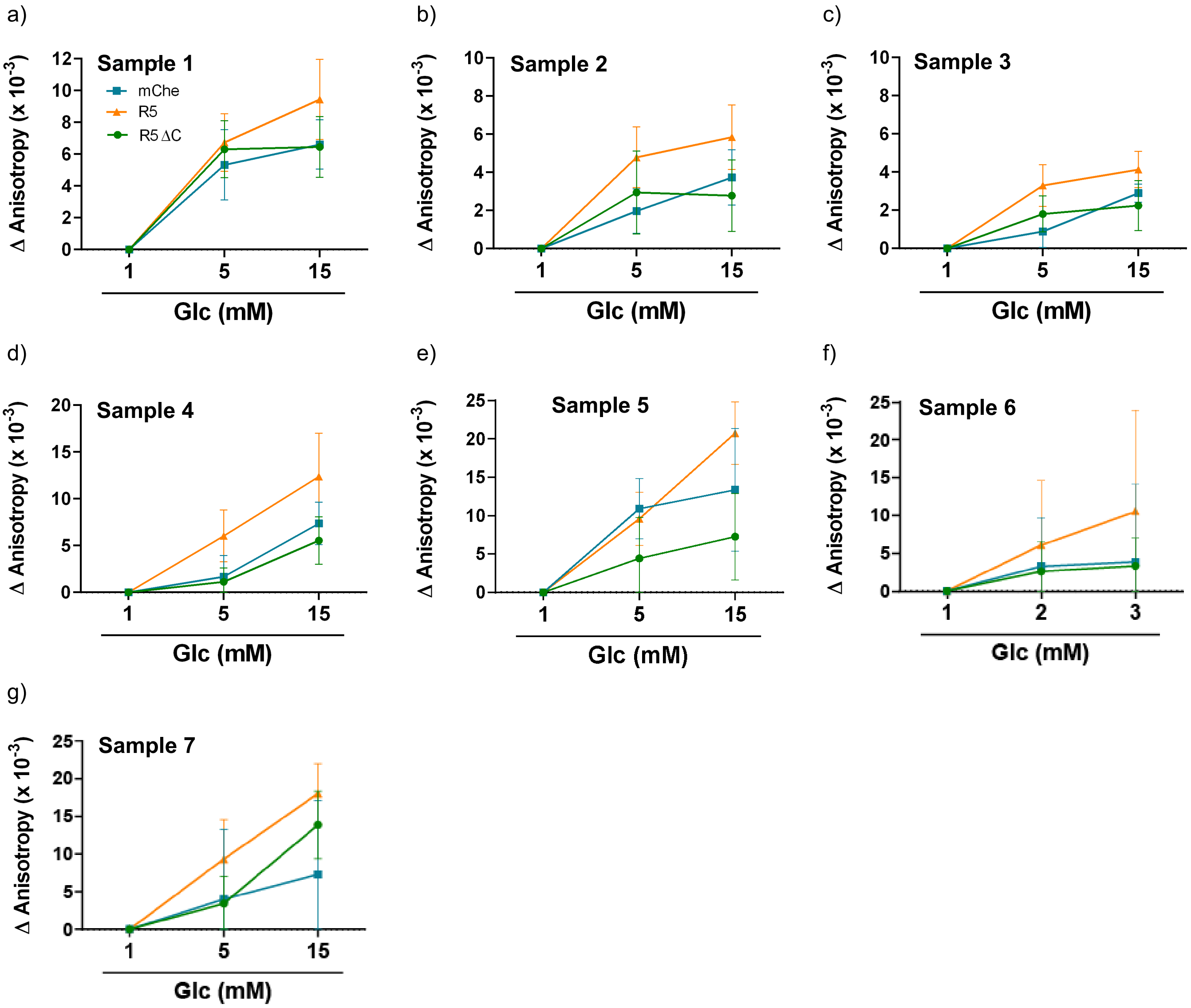

Figure S8. R5 expressing samples possess a more robust glucose dose response in seven human islet preparations. Seven separate human islets from donors of varying age (a, 45; b, 45; c, 8; d, 29; e, 42; f, 48; g, 44), sex (a, F; b, M; c, F; d, M; e, M; f, M; g, M) and BMI (a, 29.7; b, 29.7; c, 15.9; d, 26.0; e, 29; f, 27.5; g, 24.9) were dispersed to single cells and co-transduced with Apollo-NADP$^+$ sensor and either mChe, R5, or R5ΔC. Cells were sequentially stimulated with 1 mM, 5 mM, and 15 mM glucose (average data shown in Fig.7b). All samples shown individually and plotted as the mean ± SD (a-g).

S9)

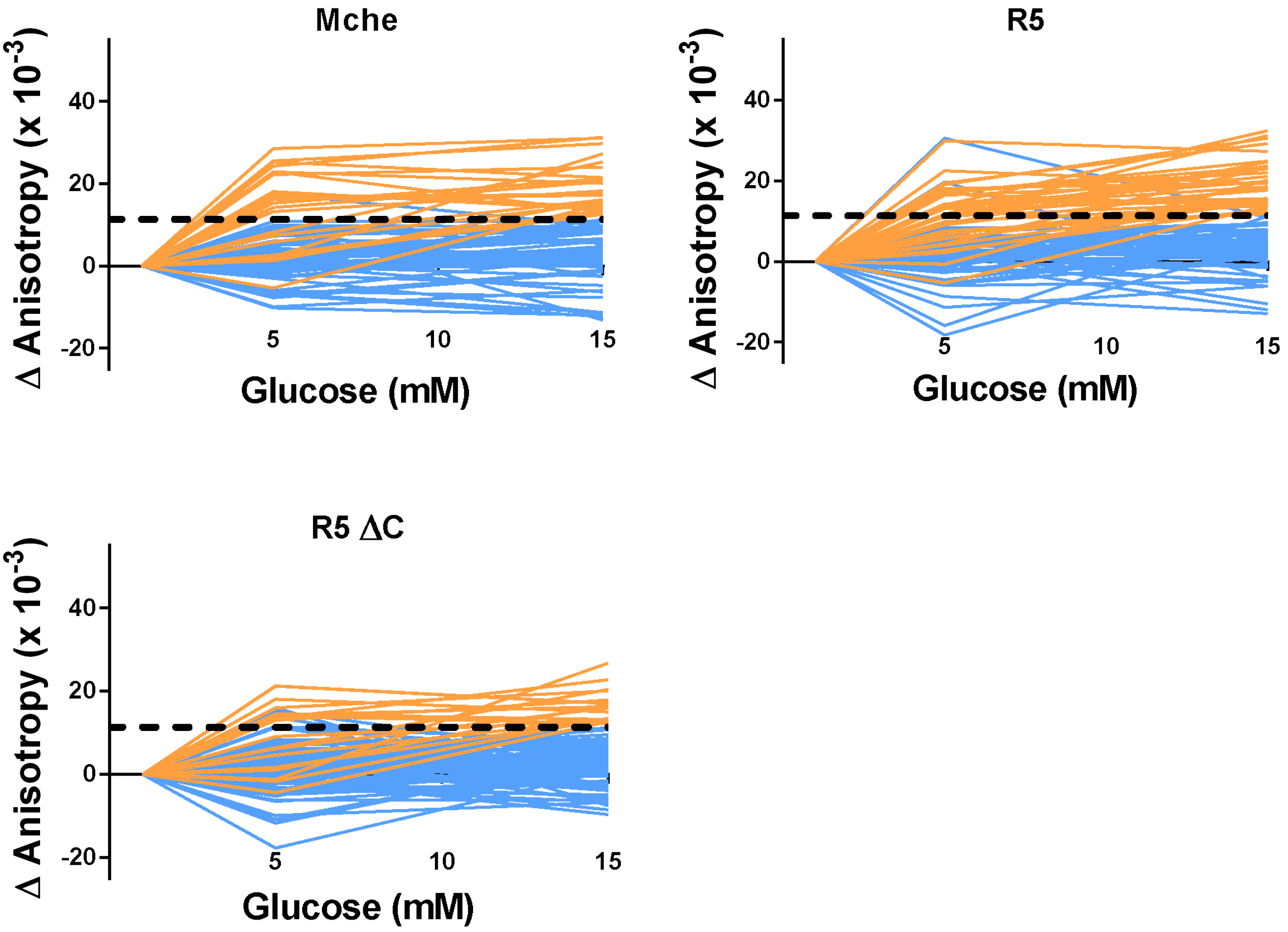

Figure S9. Glucose-stimulated NADPH response traces of human beta-cells indicating strong vs. weak responders. Human islets co-transduced with the Apollo-NADP$^+$ sensor and one of the mChe, R5, or R5ΔC constructs were exposed to a glucose dose response and the metabolic readouts were collected on a single cell basis to generate metabolic response traces. The single cell traces from each sample are grouped into strong (blue) and weak (orange) response categories based on a threshold determined by the mChe expressing control cell population (fraction of strong vs. weak responders shown in Fig. 7c).

Primer Sequence List		
Primer	Sequence	
	Forward (5'-3')	Reverse (5'-3')
FGFR5 (overexpression)	TGATCCCTGTCGAGCGTCTA	GGGACCACTTTGTCTGCCAT
FGFR5	GTCCAGTCCAGCTCCCAGTG	GTGGGACCACTTTGTCTGCC
FGFR1	CCCAACCTTGCCTGAACAAG	GCCTACGGTTTGGTTTGGTG
KLB	CAGGTATGCATGCACCAGGA	CCTTCTGATGAGGGCGGAAG
GLUT 2	CGGGGACAAACTTGGAAGGA	TGAGGCCAGCAATCTGACTA
HK1	CTGCTCACCAGGGCTACTG	CCCTTTTCTGAGCCGTCCG
GCK	GGAAGTCTGGGCTACTTCTGC	ACTCTGCCAGGATCTGCTCTA
PKM2	GTGCCACACAGATGCTGGA	CTCAGCCTCCCGAGCTATCA
FASN	GATGGAAGGCTGGGCTCTATG	AGTGTTCGTTCCTCGGAGTG
MAFA	TCGACCTGATGAAGTTCGAGG	TGGTACCCGCTCATCCAGT
UNC3	AGAAGACAAAGCTGCAACCC	AAGTAGGTGGGCATCAGCATC
CX36	TCTTGGAGAGGCTGCTGGAA	ATCATCGTACACCGTCTCCC
ITGA6	GCTTCACCCAGAACACCGTA	AAATCCCAGTCCTTCCGCTG

Table S1

Table S1. Primer sequences used in qPCR. Forward and reverse complimentary strands designed using NCBI primer-BLAST and manufactured by Integrated DNA Technologies.
